# Refining gene signatures: a Bayesian approach

**DOI:** 10.1186/1471-2105-10-410

**Published:** 2009-12-10

**Authors:** Amira Djebbari, Aurélie Labbe

**Affiliations:** 1 Knowledge Discovery Group, Institute for Information Technology, National Research Council of Canada,46 Dineen Drive, Fredericton, NB, Canada; 2 Department of Epidemiology, Biostatistics and Occupational Health,1020 Pine Avenue West, Montréal, QC, Canada; 3 Douglas Mental Health research Institute,6875 LaSalle Blvd., Montreal, H4H 1R3, QC, Canada

## Abstract

**Background:**

In high density arrays, the identification of relevant genes for disease classification is complicated by not only the curse of dimensionality but also the highly correlated nature of the array data. In this paper, we are interested in the question of how many and which genes should be selected for a disease class prediction. Our work consists of a Bayesian supervised statistical learning approach to refine gene signatures with a regularization which penalizes for the correlation between the variables selected.

**Results:**

Our simulation results show that we can most often recover the correct subset of genes that predict the class as compared to other methods, even when accuracy and subset size remain the same. On real microarray datasets, we show that our approach can refine gene signatures to obtain either the same or better predictive performance than other existing methods with a smaller number of genes.

**Conclusions:**

Our novel Bayesian approach includes a prior which penalizes highly correlated features in model selection and is able to extract key genes in the highly correlated context of microarray data. The methodology in the paper is described in the context of microarray data, but can be applied to any array data (such as micro RNA, for example) as a first step towards predictive modeling of cancer pathways. A user-friendly software implementation of the method is available.

## Background

High density arrays evaluate DNA, RNA and protein levels at the genome and proteome scale. These high throughput experiments enable the identification of some biomarkers associated with disease. For example, classification of gene expression profiles has the potential to help diagnosis, prognosis, to suggest targeted treatment and to predict response to treatment. From the machine learning point of view, it is well known that high throughput array data suffers from the curse of dimensionality where a relatively small number of samples (in the tens) compared to the number of genes (in the tens of thousands), so that we face over-fitting. Over-fitting occurs when a supervised learning algorithm adapts too well to the training data and ends up performing well on training data but not on testing data. Feature selection is one of the ways to counter over-fitting.

The goal of feature selection is to find genes (features) that best distinguish groups of instances (e.g. disease vs. normal) to reduce the dimensionality of the dataset. Several univariate statistical methods (also known as filter methods) including t-test, significance analysis of microarrays (SAM) [1], and analysis of variance (ANOVA) have been applied to select features from microarray data. In classification experiments, feature selection methods generally aim to identify relevant gene subsets to construct a classifier with good performance [2]. Features are considered to be relevant when they can predict the class: the strongly relevant features are indispensable to prediction and the weakly relevant features may only sometimes contribute to prediction.

Filter methods evaluate feature subsets regardless of the specific learning algorithm used. These methods ignore the fact that there may be redundant features (features that are highly correlated with each other and as such one can be used to replace the other) and so do not seek to find a set of features which could perform similarly with fewer variables while retaining the same predictive power [3]. For this reason multivariate methods are more appropriate. Wrappers belong to the family of multivariate methods that consider the learning algorithm as a black-box and use prediction accuracy to evaluate feature subsets [4]. Wrappers are more direct than filter methods but depend on the particular learning algorithm used. Several search algorithms can be used such as forward selection (starting with an empty set and adding features one by one) or backward elimination (starting with all features and removing them one by one). From the classification standpoint, the simple naïve Bayes classifier [5] has been shown to perform at least comparably to decision trees [6,7]. A naïve Bayes classifier contains a directed edge between the class variable and every other node and no edges between the other nodes. In other words, in naïve Bayes classifiers, the following assumptions hold: all variables are relevant to classification and other variables than the class are independent of each other.

These assumptions are often violated in many application domains and several research directions have been developed to relax either of those assumptions. One of these research direction is to allow the addition of directed edges between attributes such as tree augmented naïve Bayes [8], Bayesian network augmented naïve Bayes or general Bayesian network augmented naïve Bayes [9]. Another way to leverage the naïve Bayes assumption is selective naïve Bayes [10], where the idea is to use only a subset of features by ignoring features that reduce classification accuracy.

Within the selective naïve Bayes approaches, a maximum a posteriori (MAP) method was recently presented by [11] to select the most probable subset of variables compliant with the naïve Bayes assumption. This method introduces a compromise between the number of variables and the performance of the classifier. Our work is closely related to the approach presented by [11] but different in that we penalize for the correlation between variables in the model. We argue that this is important for high density array datasets due to their highly correlated nature, as it is well-known that genes act together in pathways [12]. We performed an extensive simulation study in order to evaluate the performance of our algorithm and then applied our approach on real cancer datasets. Our results show that we obtain either similar or better performance than other competing methods with a smaller number of genes.

## Methods

The problem we face is the following: given *K *variables (e.g. gene expressions) and a class variable *Y *(e.g. clinical outcome), we aim to select *m *variables that predict the response *Y*. In the microarray literature, this set of *m *variables is called the gene signature. In this paper, we propose a two-step approach to refining gene signatures. Specifically, our work consists of a Bayesian method for feature selection with regularization which penalizes correlation between the variables selected. The method is developed in the context of microarray data, but can be applied to any array data (such as micro RNA, for example), where the variables (e.g. gene expressions) are continuous. First, from the thousands of genes present in an array, we select the few (hundreds or less) of those associated with the class variable using some known feature selection methods, or by using some existing gene signatures. From these pre-selected genes, we then use a forward selection algorithm with a beam search in order to find the smallest and least correlated subset of variables. This search is done using a cost function derived from the posterior likelihood of a naïve Bayes classifier, including the probability of selecting a model as a prior which penalizes models containing highly correlated variables.

### Model

Let (*X*_1_,..., *X*_*K*_) be the set of *K *gene expressions (assumed to be continuous) available for each of the *N *biological samples (e.g. biopsies collected from patients), such that *X*_*k *_= (*X*_*k*1_,..., *X*_*kN*_). Let *Y *= (*Y*_1_,..., *Y*_*N*_) be the class variable one wants to predict, taking values in {1, 2}, assuming that *Y*_1 _,..., *Y*_*N *_are independent. We also note ℳ_*m *_the set of all possible sets of *m *gene expression variables chosen without replacement among the *K *gene expression variables available. Finally, we note  the set of all possible combinations of *m *variables chosen among *K *variables.

In the naive Bayes classifier setting, we note *P *(*X*_*k*_*|Y *= *y*) the conditional density distribution of *X*_*k *_given *Y *= *y *and we assume that *X*_1_,..., *X*_*K *_are all independent conditionally to the class variable *Y*.

For a given set of variables *M *∈ ℳ, the posterior probability of *Y *is

We shall assume a prior distribution on the set of variables *M *included in the model. Specifically, if model *M *is of size *m *(i.e. contains *m *variables), we set

where *P *(*M *∈ ℳ_*m*_) represents the probability to choose a set of variables of size *m *chosen among all the possible models of any size.

In this work, we wish to weigh each probability *P *(*M*) according to the amount of correlation among the variables present in *M*. In other words, if the variables from *M *are highly correlated, the probability to select this particular set of variables is low, and vice versa. Specifically, we set the probability of selecting a model *M *as an inverse function of *Cormax*(*M*), the maximum of all the pairwise correlations among the variables of *M *:(1)

In this paper, we do not claim to model the correlation structure of a signature although we acknowledge it would be interesting to do that. Note that we only measure the magnitude of the correlations, and the *Cormax *statistics was designed to answer this objective.

To compute the denominator of equation (1), it is clear that enumerating all the possible models containing the edge with maximum correlation would be computationally prohibitive. Instead, we can notice that the number of models of size *m *with maximum correlation among all pairs of variables in the model can be easily computed using the binomial coefficients from Pascal's triangle. First, let *ρ*_*ij *_be the correlation measure between (*X*_*i*_, *X*_*j*_), with *i *= 1,..., *K*, *j *= 1, ..., *K *and *i *≠ *j*. We also note the corresponding ordered correlations *ρ*^(1) ^≥ *ρ*^(2) ^≥ .... ≥*ρ*^(*K*(*K*-1)/2)^. Now, consider a Pascal triangle with (*K *- 1) rows (recalling that the first row/column of Pascal triangle is noted row/column 0th) and let us note *P*_*qr *_the entry of the triangle corresponding to row *q *and column *r*, which corresponds to the combination . We now define the vector *Z *of size *K*(*K *- 1)/2 such that we repeat the triangle coefficients of column *j *in reverse order by shifting one position each time:

Then, we have

where *Z*_*l *_is the *l*th element of *Z*. A simple example of the calculation above is presented in the Appendix. Now that we have set the probability of selecting a particular model of size *m*, we can define the prior probability of selecting a model containing *m *variables:(2)

where variables are chosen with replacement according to the same argument as described in [11].

However, one can notice that the above probability is a monotonic function increasing with the model size *m*. This implies that one would tend to prefer models with a large number of variables. In order to penalize for large models, we choose the following instead of (2)(3)

noting that (2) and (3) are symmetric. In the Bayesian framework, the posterior probability of a model *M *is evaluated as the product of the prior and the likelihood. The log-posterior probability of a model *M *is then

The middle part of this posterior probability penalizes for the correlation among variables in the model, whereas the last part penalizes for the size of the model. This means that the posterior probability of a model *M *decreases with the amount of correlation among variables in *M *and decreases with the number of variables in *M *. In cases where data are highly correlated, as it is the case in microarray data, one may want to give more weight to the correlation penalty, and by including a weight lambda, the penalty terms become:

## Results

In order to evaluate our approach, we first performed an extensive simulation study (section 3.1) and then applied our approach on real datasets (section 3.2). The goal of the simulation is to evaluate the performance of our method in the general context of feature selection with correlated variables, where we simulate data such that only a subset of the variables predicts the class. For fair comparison, we compared our approach with two other methods based on a naïve Bayes classifier: a naïve Bayes wrapper [4] and the [11] method (our proposed strategy is a direct extension of their work). Note that the wrapper searches for feature subsets by optimizing the classification accuracy and it is known as a very powerful multivariate approach. For each method, we identified whether we could successfully retrieve the original subset of variables that was simulated to predict the class. In such a way, we are able to evaluate our approach and we shall show later that we can most often recover the correct subset of genes that predict the class, even when accuracy and subset size remain the same. The Weka machine learning tool [13] was used in all the experiments described below.

### Simulations

**Figure 1:**
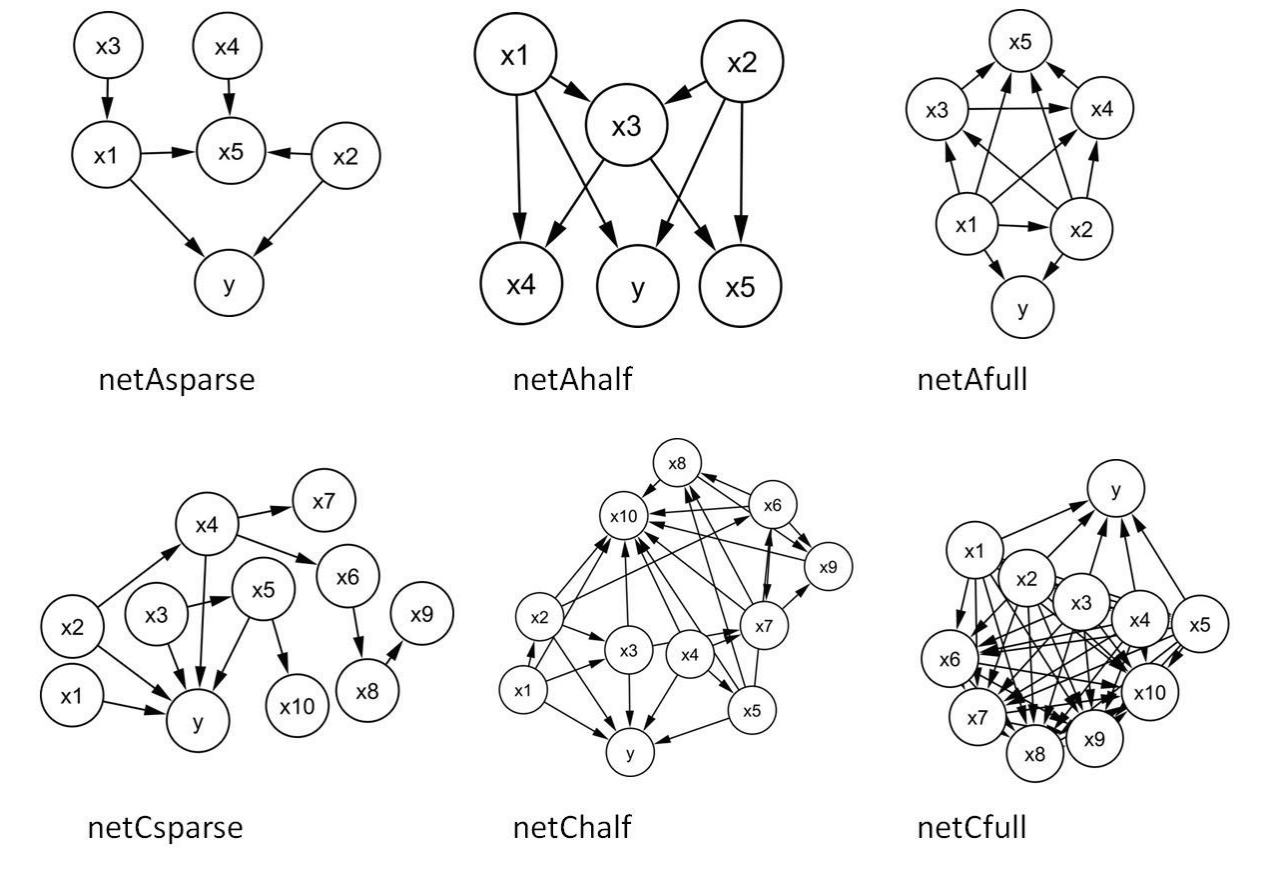
**Networks used for simulations**.

Our simulation study was designed such that only a subset of the variables simulated predicts the class outcome. Our aim is to address the following questions: i) Can we select the relevant features; ii) Can we remove redundant features; iii) Can we handle noisy data; iv) How does the number of features and instances affect the results; v) What is the impact of the penalty weight on the feature selection results. For the simulations, we supposed that a subset of variables predicts the class according to a logistic model and that other variables can be correlated with them. We simulated correlations among variables using a network representation, as shown in Figure 1. Nodes without parents were simulated according to a standardized normal distribution with mean 0. Children of those nodes were simulated using a linear regression model containing their parents with coefficients uniformly randomly chosen between 1 and 5. Finally, the class outcome *Y *in the network was simulated according to a logistic model containing only the parents of *Y *. We varied the connectivity in the networks from sparse, full or half connection (with number of edges in between sparse and full) for 2 sets of 5 and 10 variables respectively (see Figure 1). In each of these 6 cases, we generated datasets varying sizes for training (see Table 1) and 1000 instances for testing. We repeated this simulation over 100 iterations in each of the 12 situations. On each training dataset, we performed a 10 fold cross-validation. We also compared the performance of our approach with varying penalty weight lambda 1 and 100, denoted corr1 and corr100 respectively) with Boullé's method and the wrapper approach. Over the 100 iterations, we computed the number of times the correct subset of variables was found.

**Table 1:** Summary of simulated datasets

Network	Total # variables	# variables predicting the class	# instances in training dataset
Dataset A100	5	2	100
Dataset A500	5	2	500
Dataset C100	10	5	100
Dataset C500	10	5	500

#### Can we select the relevant features?

First, consider the simple case of finding 2 variables out of 5 from the dataset A100-sparse. All methods seem to perform similarly in terms of training accuracy (85.48% on average +/- 0.18% over the methods considered), testing accuracy (84.87% on average +/- 0.08%) and number of selected variables (2.54 on average +/- 0.14). However our approach finds the correct variables more often than the other methods (Table 2).

**Table 2:** Number of times (out of 100) the correct variables are found with each method for network A

Dataset	Boullé	Wrapper	Corr1	Corr100
A100-sparse	55	46	59	97
A100-half	43	40	47	98
A100-full	45	35	47	92
A500-sparse	64	37	64	97
A500-half	50	33	50	93
A500-full	48	30	55	96

#### Can we remove redundant features?

When varying the connectivity with the network of 5 variables, our approach always outperforms the others in terms of finding the correct variables. Increasing the penalty weight lambda improves the performance (Table 2). Similarly, when varying the connectivity with the network of 10 variables (Table 3), we see that it is more difficult with the small training set size to recover the correct variables (with 9 variables recovered with half connectivity or 12 variables recovered with full connectivity for dataset C100) but increasing the number of instances in training improves the performance of our approach (with 75 variables recovered with half connectivity and 84 variables recovered with full connectivity for dataset C500).

**Table 3:** Number of times (out of 100) the correct variables are found with each method for network C

Dataset	Boullé	Wrapper	Corr1	Corr100
C100-sparse	22	3	35	45
C100-half	5	2	5	12
C100-full	1	2	3	9
C500-sparse	35	13	52	98
C500-half	32	8	51	75
C500-full	34	5	52	84

#### Can we handle noisy data?

We added noise to the training sets by randomly flipping the values of 5, 10 and 20% of the class variable. Our approach outperforms the others but we note that the wrapper approach is fairly tolerant to noise (Table 4).

**Table 4:** Number of times (out of 100) the correct variables are found with each method for network C-sparse

Dataset	Boullé	Wrapper	Corr1	Corr100
C100-noise0	22	3	35	45
C100-noise5	16	3	22	27
C100-noise10	7	3	19	32
C100-noise20	6	3	12	17
C500-noise0	35	13	52	98
C500-noise5	32	11	48	97
C500-noise10	22	12	35	87
C500-noise20	15	10	27	77

#### How does the number of features and instances affect the results?

By comparing tables 2 and 3, we can see that the number of features correlated with the subset of interest greatly affects the methods ability to recover the correct subset of variables. This is true for every method. However, with increasing number of instances, our approach can find the correct subset even though the other compared methods did not.

#### What is the impact of the penalty weight *λ *on the feature selection results?

We performed the same experiments as previously with varying penalty weight *λ *(1, 10, 100, 500 and 1000) and found that increasing *λ *generally improves the results. However, increasing *λ *over 500 doesn't seem to make any difference in terms of the number of correct subsets recovered (results not shown).

### Real datasets

We also applied the proposed method to real cancer datasets. In this case, gene expressions are usually measured from biopsies collected on patients affected by a given type of cancer. For each real dataset, the data we used was normalized according to the methods in the papers describing the datasets.

The class variable *Y *may represent the cancer prognostic, for example. Note that in this case, we do not know the true set of prediction variables but can only measure classification performance. It is important to note that accuracy (ACC) is not the best measurement for model evaluation [14]. This is why we also computed the Area Under the receiver operating Curve (AUC) [15], as there is often a trade-off between misclassifications in terms of true positives (TP) or false positives (FP). We remind that the Receiver-Operator Characteristic (ROC) curve compares sensitivity and specificity directly by plotting the TP rate vs. FP rate (Fawcett, 2003). Computing the area under this curve (AUC) reduces the ROC performance to a single scalar value and it represents the probability that the classifier will rank a randomly chosen positive instance higher than a randomly chosen negative instance. We also measured sensitivity (SENS), specificity (SPEC), Positive Predictive Value (PPV), which is the proportion of True Positives (TP) over all the predicted positives, and Negative Predictive Value (NPV), which is the proportion of True Negatives (TN) over all the predicted negatives. Since models must be evaluated carefully to prevent selection bias [16], we used a 10 fold cross-validation (CV) strategy for feature selection. It is important to apply CV not only on the creation of the prediction rule but also on the feature selection otherwise a bias is introduced in the estimated error rates resulting in over-optimistic classification accuracy [17]. As a consequence, results from many studies are controversial due to methodological flaws [18]. Therefore, models must be evaluated carefully to prevent selection bias [16]. Nested CV is recommended, with an inner CV loop to perform the tuning of the parameters and an outer CV to compute an estimate of the error [19]. It is important to note that using nested CV, a different feature subset of variables is selected at each iteration. To look at the stability of the feature subsets selected at each fold, we could use the stability index proposed by [20]. However, this index is defined only to compare subsets of the same size, which is not applicable in our case. In a similar spirit, we suggest instead to look at the stability of the feature subsets by considering the number of folds where each variable was selected.

#### van't Veer dataset

We first ran our algorithm on the 70 genes signature from the van't Veer breast cancer dataset [21]. Van't Veer and colleagues investigated the problem of molecular classification of breast cancer and found a 70 genes profile also known as the Amsterdam signature to predict breast cancer prognosis. From the key observation that 70 to 80% of patients receiving chemotherapy would have survived without it, [21] identify a 70 genes signature to classify good prognosis (patients who remained free of disease for at least five years) and poor prognosis (patients who developed distant metastases within five years). The van't Veer training set contains 78 patient samples of which 34 have poor prognosis and 44 have good prognosis. In a follow-up study by [22], tumors of primary invasive breast carcinoma less than 5 cm in diameter from 295 women were examined for validation. The cohort of 295 patients studied includes 61 of the 78 patients from the previous study [21]. The Amsterdam signature assigns a given instance to good prognosis if this instance has correlation coefficient greater than 0.4 with the 70 genes profile, and assigns the instance to poor prognosis otherwise. For training, we used 77 samples instead of the original 78 as one sample had 44.5% missing value.

Note that we used preprocessed data for both van't Veer and van de Vijver datasets where the fluorescence intensities were quantified, corrected for background noise and normalized [21,22].

**Figure 2:**
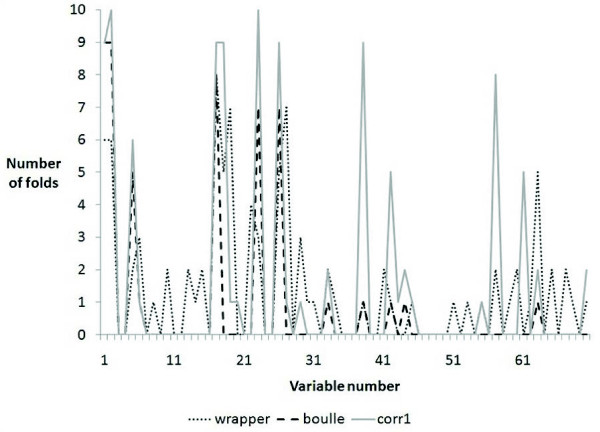
**Number of times genes were selected in 10 fold nested CV for van't Veer breast cancer dataset**.

First, we evaluated the performance of each approach with a nested 10 fold CV looking at the classification performance (note that results did not change for lambda greater than 10). We also looked at the genes selected in each fold to evaluate the consistency of the nested CV. Figure 2 shows the frequency of the genes found over the folds. It shows that our approach is more stable than the others.

We also evaluated the performance of the different methods on the independent test set from the van de Vijver dataset [22]. We considered only the 234 new tumor samples from the van de Vijver dataset (not included in the previous study) as the independent test set. Our approach finds 11 genes with at least 50% confidence in nested 10 fold CV (that means genes that were found in at least 5 of the 10 folds). Even though the wrapper and Boullé methods produce less number of genes, their sensitivity is significantly lower than with our approach (Table 5). Furthermore, our accuracy on the independent test set is not significantly different than the 70 genes in the Amsterdam signature (p = 0.6069 with McNemar test) but used 11 genes instead of 70.

**Table 5:** Classification performance of naïve Bayes algorithm on genes from van't Veer breast cancer dataset as training and independent test set of 234 samples for testing.

	Boulle	Wrapper	Corr1	Corr10	Amsterdam Signature
Average # of genes	6	8	10	11	70
ACC (%)	66.24	60.68	61.54	63.25	61.54
SENS (%)	69.57	65.22	73.91	81.16	86.96
SPEC (%)	64.85	58.79	56.36	55.76	50.91
PPV (%)	45.28	39.82	41.46	43.41	42.55
NPV (%)	83.59	80.17	83.78	87.62	90.32
AUC	0.6721	0.6200	0.6514	0.6846	0.6893

The average number of genes represents the average over the nested 10 fold CV.

#### Pomeroy medulloblastoma outcome dataset

[23] investigated the response to treatment of medulloblastomas with gene expression profiles from biopsies of 60 similarly treated patients and found a 100 genes signature.

**Figure 3:**
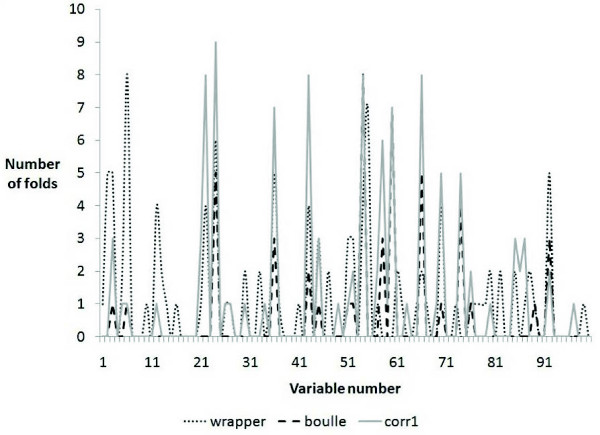
**Number of times genes were selected in 10 fold CV for Pomeroy medulloblastoma outcome dataset**.

Note that we used the preprocessed Pomeroy medulloblastoma dataset after thresholding, filtering and rescaling as described in [23]. We applied our algorithm to the Pomeroy medulloblastoma training dataset of 60 samples (39 survivors, 21 failures) to predict clinical outcome and evaluated the classification performance using a 10 fold nested CV using the original 100 genes signature. In general our prediction is slightly better than the prediction obtained using Pomeroy's 100 genes but used only 11 genes (with at least 50% confidence in nested 10 fold CV) instead of 100 (Table 6). Varying the penalty weight lambda did not alter the results. As we did before, we also looked at the stability of the subset of genes selected in each fold and found that our approach is more stable compared to others (Figure 3).

**Table 6:** Classification performance of naïve Bayes algorithm with nested 10 fold CV obtained by different methods on Pomeroy medulloblastoma outcome dataset.

	Pomeroy Signature	Wrapper	Boulle	corr1
Average # of genes	100	11.5	4.8	11
ACC (%)	73.33	71.67	61.67	75.00
SENS (%)	76.92	74.36	76.92	82.05
SPEC (%)	66.67	66.67	33.33	61.90
PPV (%)	81.08	80.56	68.18	80.00
NPV (%)	60.87	58.33	43.75	65.00
AUC	0.8168	0.8180	0.7410	0.7800

The average number of genes represents the average over the nested 10 fold CV.

#### Ramaswamy metastases dataset

[24] explored the gene expression profiles of human primary tumours and metastases. They found a 128 genes signature that best distinguish primary and metastatic carcinomas.

Note that we used the preprocessed Ramaswamy metastases dataset after rescaling as described in [24]. We applied our algorithm to the Ramaswamy training dataset (Ramaswamy et al., 2003) of 86 samples (64 tumors and 12 metastases of diverse origins) to predict metastases and evaluated the classification performance using 10 fold nested CV (Table 7). Our prediction is similar to the prediction obtained using Ramaswamy's 128 genes but used only 8 genes (with at least 50% confidence in nested 10 fold CV) instead of 128. The results did not change when increasing the penalty weight lambda past 100.

**Table 7:** Classification performance of naïve Bayes algorithm with nested 10 fold CV obtained by different methods on Ramaswamy metastases dataset

	Ramaswamy signature	Wrapper	Boulle	corr1	corr100
# of genes	128	5.9	3	8.4	8
ACC (%)	90.79	89.47	77.63	89.47	90.79
SENS (%)	92.19	95.31	87.50	95.31	95.31
SPEC (%)	83.33	58.33	25.00	58.33	66.67
PPV (%)	96.72	92.42	86.15	92.42	93.85
NPV (%)	66.67	70.00	27.27	70.00	72.73
AUC	0.9297	0.8650	0.7410	0.8740	0.9210

**Figure 4:**
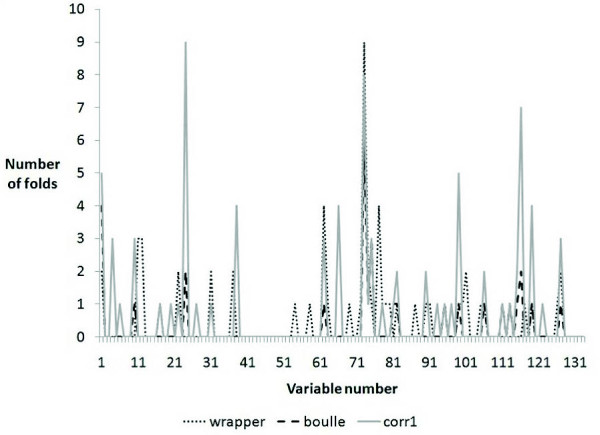
**Number of times genes were selected in 10 fold CV for Ramaswamy metastases dataset**.

We also looked at the genes selected in each fold and found that our method is more stable compared to others (Figure 4).

## Discussion and Conclusion

The main question we explore in this paper is how many genes and which genes should be selected for class prediction? We presented a Bayesian approach for feature selection with regularization to penalize for the correlation among the variables in the model. We performed an extensive simulation study where we simulated variables to predict the class in order to address the issues of redundancy, noise and scalability in terms of number of features and number of instances. In such a way we were able to evaluate the performance of our approach. We found that we can most often recover the correct genes even when the accuracy and subset size is the same than compared to Boullè's method and a wrapper approach. One of the limitations however is that small sample size can severely affect the frequency of correct subsets found highlighting the small sample size problem especially when the genes are highly correlated, as it is the case in microarray data. This finding agrees with other reports including the comparison of the removal of irrelevant and redundant features to binning [25]. It is worth noting that with enough data, unlike other methods, our approach could recover correct subsets even in fully connected networks. For the choice of the penalty weight, we tried various penalty weights and observed that the larger the penalty weight, the better the performance. However, the performance of the method did not seem to change after *λ *= 100. We then applied our method on real cancer datasets (breast cancer, medulloblastoma and metastases datasets) and showed that we can refine gene signatures with a smaller number of genes and similar or better classification performance. We also showed that the results obtained were not very sensitive to the tuning parameter *λ *.

From the biological standpoint, it is important to note that genes are known to interact with each other in pathways that bring about a particular phenotype. Genes that act together in a pathway undergo gene regulation and often have correlated expressions. If one wants to model gene interactions and infer relationships between the genes, network-based approaches are currently developing into a fruitful area of investigation. Nevertheless, the objective of our paper is not to reconstruct gene pathways derived from their correlation structure. Instead, we penalize the gene correlations in order to identify the smallest set of least correlated genes that can predict a given phenotype such as disease outcome. In this work, we ask whether we can identify crucial genes that can predict a phenotype. The interest of finding a minimal set of genes to predict the class by refining gene signatures is mainly to improve prediction with cost reduction of screening tests for diagnosis, prognosis or treatment response of future new patients in the pharmaceutical industry.

In the particular application to microarrays, how to estimate the true correlations between variables is unclear. It is known [26] that normalization procedures destroy the correlation structure of the genes on the array, and that we cannot estimate the true value of correlations between gene expressions. However, as Qiu and colleagues (2005) mentioned in their paper: "When analyzing real world biological data sets, normalization procedures are unable to completely remove correlation between the test statistics. The long-range correlation structure also persists in normalized data". In this work, we do not claim to find a unique set of genes to predict the class but that our algorithm can find one such set with least mathematically correlated genes.

In future work, we aim to extend this idea to Bayesian networks which relax the assumption of variable independence of naïve Bayes. In a Bayesian network, the Markov blanket of a class variable Y is the minimal set of variables such that Y is independent of all other variables given its Markov blanket. The Markov blanket of Y therefore probabilistically shields off Y from the rest of the network and would provide a network approach towards the predictive modeling of cancer pathways.

Finally, note that a user-friendly software implementation of the method proposed in this paper is available at http://www.medicine.mcgill.ca/epidemiology/Labbe/Publications.html.

## Authors' contributions

AD and AL conceived of the study and wrote the manuscript. AD devised, implemented and tested the algorithms and evaluated the results. AL contributed to the experimental design and also to the statistical analysis of the results. Both authors read and approved the final manuscript.

## Appendix

We provide here an example of how to compute the denominator of equation (1) in Section 2.1 when *K *= 5 and *m *= 3, i.e:(4)

This is equivalent to compute the sum over all possible models of size 3 (chosen among 5 variables) of the maximum of the pairwise correlations in each model. We denote the 5 variables {1, 2, 3, 4, 5} and assume without loss of generality that the ordered correlations are *ρ*_12 _≥ *ρ*_13 _≥ *ρ*_14 _≥*ρ *_15 _≥ *ρ*_23 _≥ *ρ*_24 _≥ *ρ*_25 _≥ *ρ*_34 _≥ *ρ*_35 _≥ *ρ*_45_. If one could list all possible models of size 3 chosen among 5 variables, equation (4) could be written as:

To compute the formula above, we first construct a Pascal triangle with *K *- 1 = 4 rows:

By looking at the (*m *- 2) = 1st column of the triangle (recalling that the first column is noted 0), we define

Then, Equation (4) is equal to:
